# Same same, but different: growth responses of primary and lateral roots

**DOI:** 10.1093/jxb/eraa027

**Published:** 2020-01-20

**Authors:** Sascha Waidmann, Elizabeth Sarkel, Jürgen Kleine-Vehn

**Affiliations:** 1 Department of Applied Genetics and Cell Biology, University of Natural Resources and Life Sciences (BOKU), Vienna, Austria; 2 INRA, France

**Keywords:** Lateral root, nutrients, plant hormones, primary root, root system architecture

## Abstract

The root system architecture describes the shape and spatial arrangement of roots within the soil. Its spatial distribution depends on growth and branching rates as well as directional organ growth. The embryonic primary root gives rise to lateral (secondary) roots, and the ratio of both root types changes over the life span of a plant. Most studies have focused on the growth of primary roots and the development of lateral root primordia. Comparably less is known about the growth regulation of secondary root organs. Here, we review similarities and differences between primary and lateral root organ growth, and emphasize particularly how external stimuli and internal signals differentially integrate root system growth.

## Teaser

We highlight the similarities and differences in primary and lateral root growth, focusing on the differential impact that phytohormones and environmental cues have on these.

## Introduction

The root system’s extensive epidermal surface facilitates the interphase between the plant and soil environment, supplying the plant with water and nutrients (Zürcher and Muller, 2016). A robust root system is key for plant productivity and hence an important agronomical trait (Lynch, 1995). Root systems are comprised of primary roots (PRs), which anchor the system into the soil by growing downwards, and lateral/secondary roots (LRs), which explore the soil through environmentally responsive radial growth and determine the overall root system size.

**Fig. 1.**
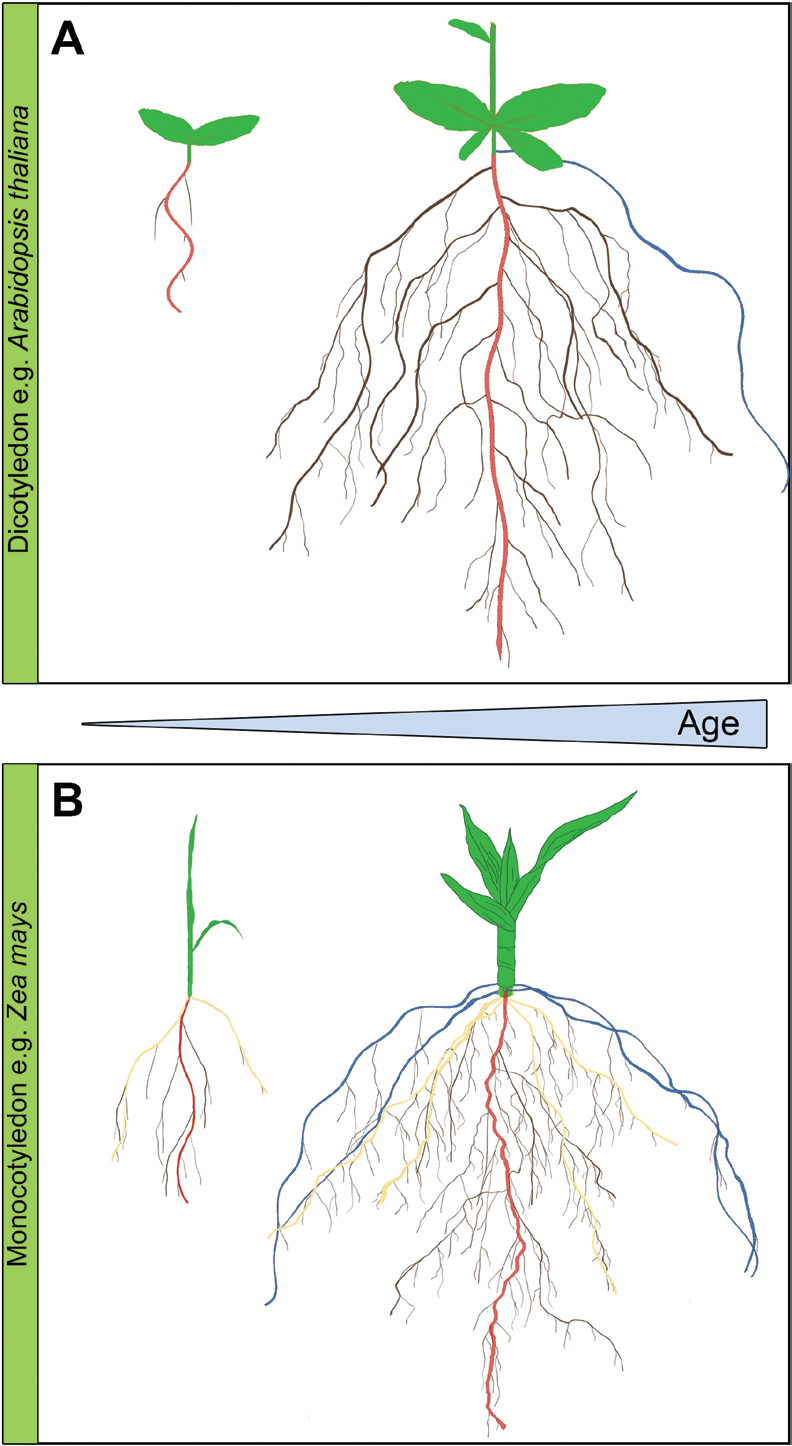
The RSA of dicots and monocots changes over time. (A) A typical taproot system of dicots (e.g. *Arabidopsis thaliana*), consisting of embryonic primary root (PR, red) and branching lateral roots (LRs, brown). Ultimately, the LRs will undergo higher order branching to form secondary and tertiary LRs. Adventitious roots (ARs, blue) form at the shoot–root junction. (B) The fibrous monocot root system of *Zea mays* consisting of an embryonic primary root (PR, red), embryonic seminal roots (SRs, yellow), which originate close to the top of the PR, crown roots (CRs, blue) that originate from the stem, and LR branching (brown) from the PRs, SRs, and CRs. Ultimately, the LRs will undergo higher order branching to form secondary and tertiary LRs.

In both monocots and dicots, the PR develops from an embryonically formed meristem (De Smet *et al.*, 2010) and is the first organ to emerge from the germinating seed. Lateral root primorida (LRPs) originate in monocotyledons and dicotyledons from founder cells in the pericycle, which is the outermost layer of the vascular cylinder (Fig. 1A; Dubrovsky *et al.*, 2000). Interestingly, in most fern species, LRs are derived from the endodermis, while in some water plants they are derived from both the pericycle and endodermis (Lin and Raghavan, 1991; Clowes, 1992; Hou and Hill, 2004). Each LRP undergoes coordinated cell division and cell expansion, leading to LR emergence and meristem activation. Many hormonal and environmental cues affect LRP priming, initiation, and development. Understanding these cues is a highly vibrant research area and we would like to refer to several excellent reviews on this topic (Fukaki and Tasaka, 2009; Péret *et al.*, 2009; Lavenus *et al.*, 2013; Motte *et al.*, 2019). In this review, we will focus on the further development of already emerged LRs.

In later stages of development, LRs themselves undergo branching to form tertiary and subsequently even higher order LRs (Osmont *et al.*, 2007). Although PR and LR development in monocots, such as rice (*Oryza sativa*) and maize (*Zea mays*), seems largely similar to their development in dicots, the overall root system architecture (RSA) is more complex in monocots (Fig. 1B). In later developmental stages, a very fibrous root system is built including embryonic seminal roots (SRs) and various post-embryonic LRs. They later dominate the root system of the adult plant and take over most of the water and nutrient uptake (Hochholdinger, 2004) (Fig. 1B). Additionally, plants readily develop adventitious roots (ARs), which are post-embryonic shoot-borne roots. In monocots, they are sometimes referred to as crown roots when formed below the soil surface and as brace roots when formed above the soil surface (Kovar and Kuchenbuch, 1994). In this review we will mainly focus on LRs of PRs and refer the interested readers to other excellent reviews on ARs (Bellini *et al.*, 2014; Ge *et al.*, 2019; Lakehal and Bellini, 2019).

RSA varies widely both between and within plant species. This natural variation underlines the importance of the RSA for plant adaptation to the environment (Cannon, 1949; Lynch, 1995; Osmont *et al.*, 2007; Hodge *et al.*, 2009; McNear, 2013; Rellán-Álvarez *et al.*, 2015). Because various conditions influence RSA by modulating the angle, rate, and type of individual roots, these aspects of RSA are important target traits for plant breeders (de Dorlodot *et al.*, 2007; Orman-Ligeza *et al.*, 2014). LRP development, LR emergence, organ growth, and periodic branching of higher order LRs are the main processes that increase the size of the root system (Hodge *et al.*, 2009; De Smet *et al.*, 2012). Similar to LRP development, the process and environmental regulation of LR emergence through the overlaying tissues has also been extensively reviewed, mainly focusing on Arabidopsis (Nibau *et al.*, 2008; Bennett and Scheres, 2010; Lavenus *et al.*, 2013; Tian *et al.*, 2014; Motte *et al.*, 2019).

Increasing evidence from several model plants suggests that PRs and LRs display distinct growth programmes modulated by unique molecular signals. In this review, we outline the similarities and differences between PR and LR organ growth (elongation) in response to external stimuli, such as nutrients and other environmental cues, as well as developmentally programmed signals, such as phytohormones.

## Plant hormones are key players in differential PR and LR growth

Plant hormones coordinate root growth in response to developmental (internal) and environmental (external) cues. Here we mainly focus on auxin, cytokinin (CK), abscisic acid (ABA), brassinosteroids (BRs), ethylene, gibberellin (GA), salicylic acid (SA), and jasmonate (JA), and their specific synthesis and signal transduction pathways. Extensive crosstalk between these hormone signalling pathways defines the development and growth of LRs and PRs. In the following, we will discuss how these hormones and their interactions determine PR and LR growth.

### Auxin’s dual impact on root organ growth

In addition to its central role in PR and LR initiation (reviewed in Péret *et al.*, 2009; Overvoorde *et al.*, 2010), auxin plays a role in defining organ growth rates. Auxin is synthesized in the aerial plant organs, mainly in the shoot apex and young developing leaves (Ljung et al., 2001, 2005) and is transported in the vascular cylinder to the root tip. Additionally, auxin is produced in the root (Chen *et al.*, 2014) and this local biosynthesis is crucial for meristematic activity (Brumos *et al.*, 2018). Auxin is here redirected shootward through epidermal and cortical cells. The current model of auxin flux in the root incorporates the polarized localization of various auxin transporters in the root to facilitate this path of auxin flux through the root. Root growth responses interpret fluctuations in auxin input, which can be modulated by auxin synthesis or transport, into cellular or organ elongation outcomes. Here, we will address how cellular growth outcomes modulated by auxin levels differ between PRs and LRs, and how these differential responses may be regulated by changes in auxin transport or cellular elongation.

Very low auxin concentrations promote PR elongation in Arabidopsis and maize (Gougler and Evans, 1981; Pilet, 1981). Conversely, higher concentrations inhibit PR elongation in several plant species (Thimann, 1939; Chadwick and Burg, 1967; Scott, 1972; Pilet, 1981; Pilet and Saugy, 1987; Debi *et al.*, 2003; Rahman *et al.*, 2007). Auxin has been recently shown to inhibit root growth via a non-transcriptional branch of the canonical auxin receptor TRANSPORT INHIBITOR RESPONSE 1 (TIR1) (Fendrych *et al.*, 2018).

Little is known about the direct effects of auxin concentration on cell division and elongation in LRs. When young Arabidopsis seedlings were transferred to medium containing auxin, the elongation of LRs was inhibited (Debi *et al.*, 2003; Ivanchenko *et al.*, 2010; Giehl *et al.*, 2012). Conversely, Muday and Haworth (1994) reported that auxin promotes the elongation of LRs in tomato (*Solanum lycopersicum*). This could indicate that there are diversified responses to auxin input in LRs of different species.

Phenotypic analysis of auxin-related mutants may provide further insight into the mechanisms by which endogenous modulation of auxin levels affects growth outcomes in PRs and LRs. The so-called *aberrant lateral root formation* (*alf*) mutants display a wide range of lateral root phenotypes (Celenza *et al.*, 1995). ALF1/SUR1 (SUPERROOT1) is the C-S lyase in glucosinolate biosynthesis. In constitutively active *alf1/sur1* mutants, there is an overproduction of the endogenous auxin indole-3-acetic acid (IAA) (Mikkelsen *et al.*, 2004). Intriguingly, though PR growth is largely normal in the *alf1/sur1* mutant, there is substantial excess LR growth (Boerjan *et al.*, 1995; Celenza *et al.*, 1995). Unfortunately, the causal gene for the *alf3-1* mutant phenotype has not yet been mapped. Overall, these results suggest that LRs and PRs execute differential growth responses to endogenous auxin levels. Future work is necessary to elucidate the signalling, synthesis, and transport mechanisms responsible for these differential growth outcomes.

Dynamic relocalization of auxin transporters can modulate auxin flux to facilitate alterations in growth. The main plasma membrane-located auxin transporters are the PIN-FORMED (PIN) proteins (Luschnig *et al.*, 1998; Gälweiler *et al.*, 1998), the ARABIDOPSIS THALIANA ATP-BINDING CASSETTE B (ABCB) subfamily transporters (Noh *et al.*, 2001), and the AUXIN RESISTANT1/LIKE AUX1 (AUX1/LAX) uptake permeases (Maher and Martindale, 1980; Okada and Shimura, 1990; Bennett *et al.*, 1996; Parry *et al.*, 2001). PINs redundantly regulate PR growth (Billou *et al.*, 2005; Vieten *et al.*, 2005), but it is unknown whether they have distinct contributions to growth rates in LRs. Notably, while all canonical PINs are expressed throughout PR development, some PIN genes show transient and stage-dependent expression in LRs (Rosquete *et al.*, 2013; Ruiz Rosquete *et al.*, 2018).

In addition to the PIN family of auxin transporters, the ABCBs 1, 4, 14, 15, and 19 are root-expressed intercellular auxin transporters (Cho and Cho, 2013). Notably, ABCB19/MDR1/PGP19 loss of function does not affect PR growth or LRP development (Wu *et al.*, 2007), but strongly impaired elongation rates in emerged LRs. This relates to distinct acropetal auxin transport in PRs and LRs (Wu *et al.*, 2007).

AUX1/LAX proteins are specifically required for the basipetal transport of auxin through the outer root cell layers (Rashotte *et al.*, 2000; Marchant *et al.*, 2002). Most studies did not detect differences in PR elongation of the wild type and the *aux1* mutant (Růzicka *et al*., 2007; Ivanchenko *et al.*, 2010; Lehotai *et al.*, 2012; Thole *et al.*, 2014; Street *et al.*, 2016), but Li *et al.* (2016) reported increased PR elongation in *aux1* mutants, particularly in alkaline stress conditions. AUX1 plays a prominent role in LRP development and LR emergence (Hobbie and Estelle, 1995; Casimiro *et al.*, 2001; Marchant *et al.*, 2002). Little is known about the role of AUX1 in emerged LRs, but LR length in *aux1* mutants is similar to that in the wild type (Linkohr *et al.*, 2002; Giehl *et al.*, 2012). These data suggest that AUX1 does not affect elongation in PRs or LRs under normal growth conditions but that it might modulate PR and LR elongation during environmental stress.

In summary, the phytohormone auxin fulfils multiple roles throughout the development and growth of PRs and LRs. While the responsiveness in PRs and LRs to exogenously applied auxin seems similar, current evidence suggests that endogenous transport and the nuclear signalling rate of auxin are distinct in PRs and LRs. Further work is necessary to better understand the differences in auxin responsiveness of PRs and LRs and how these may be modulated by distinct transport, nuclear signalling, and cell physiology regulatory mechanisms. Moreover, it remains unclear whether cellular auxin responses (e.g. meristematic activity and elongation) are conserved among PRs and LRs.

### Cytokinin sensitivity depends on the developmental stage of lateral roots

CK and auxin interact both synergistically and antagonistically during plant development. Like auxin, exogenously applied CK inhibits organ growth of the PR in Arabidopsis (Werner *et al.*, 2001; Werner, 2003; Mason, 2004; Li *et al.*, 2006; Laplaze *et al.*, 2007; Šimášková *et al.*, 2015), maize (Márquez, 2019), and rice (Kudo *et al.*, 2012). Mechanistically, CK negatively regulates quiescence centre (QC) specification (Zhang *et al.*, 2013) and reduces the number of cells in the meristem (Beemster and Baskin, 2000; dello Ioio *et al.*, 2008). The QC is a small group of rarely dividing cells promoting the stem cell status in the root and hence has central importance for providing various cell types (van den Berg *et al.*, 1997; Drisch and Stahl, 2015). CK interferes with auxin signalling in the transition zone, thereby regulating the onset of cellular division, decreasing meristem size (Beemster and Baskin, 2000; Werner, 2003; Li *et al.*, 2006; Šimášková *et al.*, 2015; Di Mambro *et al.*, 2017). Similarly, several publications report that the elongation of LRs is inhibited by CK in Arabidopsis (Werner *et al.*, 2001; Li *et al.*, 2006; Miyawaki *et al.*, 2006), but the cellular mechanism largely remains to be addressed. Additionally, exogenous application or endogenous increase in CK asymmetrically reduces cell proliferation in the meristematic zone and inhibits cell elongation in the elongation zone, thereby determining angular growth in young LRs (Waidmann *et al.*, 2019). However, the interaction between CK and auxin signalling in this response, and whether it mirrors the interaction that occurs in PR growth regulation, remains to be elucidated. Intriguingly, a recent study in maize (Márquez, 2019) showed that the CK effect on LR growth is concentration and tissue dependent, revealing that low CK levels only reduced LR length in the most apical/proximal region of the root system, while at high concentrations CK inhibits the LR elongation along the entire root system but with a more pronounced effect in the apical zones (Márquez, 2019). Hence, the impact of CK on the elongation of LRs could be distinct in developmentally younger and older lateral roots.

It would be interesting to assess how CK signalling, metabolism, transport, and/or distribution mechanisms are distinct in PRs and LRs. In addition, the possibly distinct contributions of environmental stimuli to CK signalling in PRs and LRs remain to be addressed.

### Lateral roots are highly sensitive to abscisic acid

Though ABA is mainly known for its role in seed germination and responses to water shortage (Yoshida *et al.*, 2019), it also regualtes root growth. High ABA concentrations reduce PR length in Arabidopsis (Chen *et al.*, 2012; Duan *et al.*, 2013; Thole *et al.*, 2014), medicago (Liang and Harris, 2005; Liang *et al.*, 2007), maize (Pilet and Saugy, 1987), and rice (Tseng *et al.*, 2013; Liu *et al.*, 2015). In Arabidopsis, some evidence suggests that the effect of ABA on PR length is dose dependent (Ephritikhine *et al.*, 1999; De Smet *et al.*, 2003; Barberon *et al.*, 2016). High ABA concentrations act via an ethylene-dependent pathway to inhibit cell division in the QC and the proximal part of the Arabidopsis root meristems (Newton, 1977; Robertson *et al.*, 1990; De Smet *et al.*, 2003; Zhang *et al.*, 2010) by suppressing transcription of cell cycle genes such as CYCLIND3;1 (CYCD3;1) and CYCLIN-DEPENDENT KINASE B1;1 (CDKB1;1) (De Smet *et al.*, 2003). On the other hand, low ABA concentrations promote root growth via an ethylene-independent pathway that requires auxin signalling and PIN2-dependent auxin transport (Li *et al.*, 2017). Additionally, low ABA concentrations act by both promoting QC quiescence and suppressing the differentiation of stem cells and their daughters (Nishimura *et al.*, 2007).

The growth responses of LRs are more sensitive to ABA signalling than those of PRs. Low concentrations of ABA show a quantitatively stronger impact on LR elongation when compared with the PR (De Smet *et al.*, 2003; Xiong *et al.*, 2006; Liang *et al.*, 2007; Chen *et al.*, 2012; Duan *et al.*, 2013). It is unknown why LRs are highly sensitive to ABA, but it probably involves, like in the PR, the down-regulation of cell cycle genes, ultimately arresting the root meristem (De Smet *et al.*, 2003). However, even in the case of persistent stress, LR growth eventually recovers from the ABA-induced inhibition (Zhao *et al.*, 2014).

These data suggest that RSA can be fine-tuned in response to fluctuations in ABA levels. Specifically, increased LR sensitivity to low levels of ABA signalling may allow restructuring of the root system to promote larger root systems that can better access soil water stores during drought conditions.

### Sensitivity to brassinosteroid, ethylene, gibberellin, salicylic acid, and jasmonate is distinct in lateral and primary roots

Root growth in response to endogenous and environmental cues is regulated by a diverse array of hormones in addition to auxin, CK, and ABA. Several of these hormones, like auxin, have concentration-dependent effects on PR elongation. In Arabidopsis, low concentrations of either BRs or SA promote PR elongation, while increased concentrations inhibit it. For BRs, this effect is conserved between several plant species (Roddick *et al.*, 1993; Clouse *et al.*, 1996; Müssig *et al.*, 2003; Kartal *et al.*, 2009). However, the effect of SA concentration is reversed in maize, rice (Kusumi *et al.*, 2006), soybean (Gutiérrez-Coronado *et al.*, 1998), *Pinus patula* (San-Miguel *et al.*, 2003), and *Catharanthus roseus* (Echevarría-Machado *et al.*, 2007); low SA concentrations induce PR growth.

These hormones probably act by modulating the balance between cell elongation and cell division in the meristem. BR maintains this balance and preserves QC identity by inhibiting or promoting differentiation of distal columella stem cells in a concentration-dependent manner (González-García *et al*., 2011; Hacham *et al.*, 2011; Fàbregas *et al.*, 2013). Interestingly, in the transition–elongation zone of the root, BRs promote cell elongation, while in the QC and surrounding cells, BR inhibits cell division (Chaiwanon and Wang, 2015; Vragović *et al.*, 2015). Low SA has a similar effect with a distinct mechanism; at concentrations below 50 µM, SA induces distal meristem enlargement by affecting auxin transport proteins, leading to an auxin accumulation in the PR (Pasternak *et al.*, 2019). On the other hand, high SA concentrations inhibit cell proliferation and elongation in the PR (Pasternak *et al.*, 2019).

Unfortunately, little is known about the role of BRs or SA on LR elongation. Only a few studies showed that BRs can enhance the elongation of LRs (Kartal *et al.*, 2009; Vercruyssen *et al.*, 2011). Whether BRs have the same dual function in the meristem of LRs as in PRs and if the response pathway works similarly in both root types remains unknown. Interestingly, application of SA in femtomolar concentrations increased root biomass due to enhanced LR growth in *C. roseus* (Echevarría-Machado *et al.*, 2007). Together with the enhanced growth of ARs, SA could promote the growth of a shallower root system, helping the plant further explore the surrounding soil. Further work will be necessary to examine how the dual effects of BRs and SA influence physiological responses to local soil conditions.

Current studies have not detected concentration-dependent effects of ethylene, JA, or GAs on PR elongation. Ethylene and JAs both negatively regulate PR growth, while GA enhances it.

First, application of ethylene or its precursor ACC (1-aminocyclopropane-1-carboxylic acid) inhibits the elongation of PRs in monocots and dicots (Le *et al.*, 2001; Swarup *et al.*, 2005; Růzicka *et al*., 2007; Ma *et al.*, 2013; Li *et al.*, 2015; Barberon *et al.*, 2016; Qin *et al.*, 2017). Ethylene and ACC negatively regulate root meristem size and overall PR length in an auxin-dependent manner (Růzicka *et al*., 2007; Alarcón *et al.*, 2014; Street *et al.*, 2015; Chen *et al.*, 2018). Interestingly, an additional auxin-independent pathway inhibits cellular elongation of epidermal cells in the PR at low ethylene concentrations (Růzicka *et al*., 2007; Alarcón *et al.*, 2014). Similarly, JAs inhibit the growth of the PR in Arabidopsis (Staswick *et al.*, 1992; Thines *et al.*, 2007; Chung and Howe, 2009; Mosblech *et al.*, 2011; Shyu *et al.*, 2012; Monte *et al.*, 2014; Thatcher *et al.*, 2016), rice (Hazman *et al.*, 2015), tomato (Tung *et al.*, 1996), and sunflower (Corti Monzón *et al.*, 2011) by inhibiting cell division and elongation (Tung *et al.*, 1996; Raya-González *et al.*, 2012; Gasperini *et al.*, 2015).

Very few studies addressed the influence of ethylene on LR organ growth. Although ethylene responses in the PR and LR appear similar (Tian *et al.*, 2009; Ramaiah *et al.*, 2014), an ethylene-sensitive reporter line suggests different molecular responses in PRs and LRs under high nitrate conditions (Tian *et al.*, 2009), enabling a specific response to environmental cues. On the other hand, JAs have an intriguing dual effect on LR elongation; in Arabidopsis and sunflower LRs, low JA promotes, while high JA inhibits elongation (Corti Monzón *et al.*, 2011; Raya-González *et al.*, 2012). Further work is necessary to understand the mechanism of this dual response to JAs.

While exogenous application of ethylene has no obvious effect on root elongation (Tanimoto, 1987, 1988, 1994; Butcher *et al.*, 1990; Du *et al.*, 2017), application of the GA inhibitor paclobutrazol (PAC) resulted in a reduced PR growth rate (Ubeda-Tomás *et al*., 2009), similar to GA-deficient mutants in Arabidopsis (Fu and Harberd, 2003; Ubeda-Tomás *et al*., 2009; Achard *et al.*, 2009). These results suggest that, GAs act in very low concentrations in the root to establish the growth-relevant arrangements of cortical microtubules and cell polarity (Baluška *et al.*, 1993). GA is important for controlling the size of the meristem and mature cell length by promoting mitotic activity in the root meristem (Ubeda-Tomás *et al*., 2009; Achard *et al.*, 2009).

While nothing is known about the effects of GAs on LR elongation in Arabidopsis, in Populus GA induces a stronger inhibition of LR organ growth when compared with the PR, presumably allowing faster stress responses in LRs (Gou *et al.*, 2010). GA signalling may enable integration of aerial and root development, reducing aerial plant growth and promoting the exploration of the root system under unfavourable growth conditions (Gou *et al.*, 2010).

In summary, the hormone effects on elongation via modulation of cell division and differentiation are more frequently understood in PRs than in LRs. Future work should examine whether the growth response to each hormone is shared between LRs and PRs across species and identify the mechanisms by which the responses are conserved or diverged. In particular, close examination of the concentration-dependent dual effects of hormones may reveal mechanisms by which PR and LR growth are tailored responses to environmental stressors.

## Root responses to environmental cues

Mineral nutrients are crucial for all aspects of plant growth and development. In most plant species, these nutrients are almost solely recruited from the soil. Most nutrients are not uniformly distributed throughout different soil layers, but rather form vertical and horizontal gradients (Jackson and Caldwell, 1993; Farley and Fitter, 1999; Lark *et al.*, 2004). In addition, several nutrients display higher mobility in the soil than others (Bray, 1954; Thomas, 1970). Hence, for efficient nutrient mining, plants need to modulate RSA in response to local soil conditions. Additionally, the structure and compactness of the soil can influence the RSA of plants (reviewed in Nawaz *et al.*, 2012). In the following subsections, we first discuss how LR and PR growth is regulated in response to fluctuations in nutrient availability. Then, we highlight how osmotic stress, temperature, light, and gravity define LR expansion and how this may differ from the PR.

### Nitrogen sources prominently define growth of lateral roots

The availability of nitrogen (N) to produce amino acids and proteins is one of the key requirements for growth and plays a major role in defining plant productivity. N is primarily taken up through the root system in its inorganic (ammonium and nitrate) forms but can also be taken up in its organic (urea, amino acids, peptides) forms. In several plant species, different responses to nitrate and ammonium have been described.

Ammonium inhibits the growth of PRs in several plant species (Tian *et al.*, 2009; Rogato *et al.*, 2010; Liu *et al.*, 2013), while LR elongation and branching is stimulated by increased local supply of ammonium in barley (*Hordeum vulgare*) and Arabidopsis (Drew, 1975; Lima *et al.*, 2010; Rogato *et al.*, 2010). This effect is most probably due to the increased expression of the AMMONIUM TRANSPROTER (AMT) in LRs, which provides enhanced transport capacities (Yuan *et al.*, 2007; Lima *et al.*, 2010).

In contrast to ammonium, the response of roots to nitrate is more complex. High local concentrations of nitrate inhibit the elongation of LRs but have no effect on the growth of PRs (Zhang and Forde, 1998; Zhang *et al.*, 1999; Tian *et al.*, 2009). Conversely, low local nitrate concentrations (>1 mM) show strong stimulatory effects on LR elongation (Zhang and Forde, 1998; Zhang *et al.*, 1999; Linkohr *et al.*, 2002; Saito *et al.*, 2014) and again have little to no influence on the length of the PR (Zhang and Forde, 1998). On the other hand, l-glutamate, another source of N, inhibits the growth of PRs and stimulates the elongation of LRs (Walch-Liu *et al.*, 2006). These data suggest that local nitrate levels regulate LR elongation, allowing dynamic RSA regulation in response to soil nitrate availability.

The different responses of PRs and LRs in nitrate excess and deficiency is most probably due to crosstalk between nitrate transporters and downstream effects on hormone signalling. NITRATE TRANSPORTER/SENSOR 1.1 (NRT1.1), but not NRT1.2, is also able to transport auxin and has been shown to transport auxin to the tip of LRs under low N availability to promote growth (Krouk *et al.*, 2010; Mounier *et al.*, 2014). Moreover, high levels of nitrate promote the expression of CLE peptides that activate CLAVATA1 (CLV1) signalling, blocking the elongation of LRs and inducing the expression of NRT1.1 and NRT1.2 but having no effect on PRs (Araya *et al.*, 2014). In addition, expression of the AGAMOUSE-LIKE21 (AGL21) transcription factor is induced under low nitrate conditions, modulating downstream expression of auxin biosynthesis genes to stimulate LR but not PR elongation (Yu *et al.*, 2014). BR signalling regulates PR growth responses to low N availability (Jia *et al.*, 2019), but a contribution to LR organ growth was not assessed.

In summary, low local concentrations of N sources promote growth and branching of LRs. This local response is likely to enable the plant to mine more N resources in the soil, tailoring growth in response to N availability. Intriguingly, different N sources elicit opposite LR growth responses: ammonium stimulates LR branching and elongation, while high nitrate causes inhibition. This suggests that root growth may be fine-tuned to the exact local N make-up. Future work examining the molecular and hormone crosstalk signals that regulate these opposite LR growth responses may uncover physiological mechanisms by which the local N composition modulates RSA. Additionally, future work should examine how local N make-up might regulate RSA by determining LR angle. These questions are particularly relevant because it has been shown that plants with a steep and deep root system perform better under low N conditions (Lynch, 2013; Zhan and Lynch, 2015; Colombi and Walter, 2017; Roychoudhry *et al.*, 2017); thus, understanding the molecular control of LR growth by local N sources would inform development of agricultural technology to facilitate robust growth responses to N deficiency.

### Phosphate sensitivity of lateral roots is age dependent

Inorganic phosphate (Pi) is a major component of nucleic acids, phospholipids, ATP, and other molecules, and is therefore crucial for plant growth and development. Because phosphate is the only source of phosphorus in the soil, RSA is highly responsive to phosphate starvation. Pi is immobile in soil, resulting in an uneven distribution and low accessibility by plant roots. Additionally, Pi can be bound by soil components or converted to organic phosphate (Yevdokimov *et al.*, 2016), further reducing the amount of plant-available Pi in the soil (Johnston *et al.*, 2014).

The response of roots to low Pi varies strongly between different species and genotypes. Numerous studies have shown a reduction in PR growth in the Arabidopsis Columbia (Col-0) accession during Pi starvation (Williamson *et al.*, 2001; Linkohr *et al.*, 2002; López-Bucio *et al.*, 2002; Sánchez-Calderón et al., 2005, 2006). However, several studies using other Arabidopsis accessions suggested the existence of several diversified strategies in response to low phosphate (Chevalier *et al.*, 2003; Reymond *et al.*, 2006; Kawa *et al.*, 2016). Using quantitative trait locus (QTL) mapping or a genome-wide association study (GWAS), several putative gene candidates were identified, which may control PR growth under low Pi conditions (Reymond *et al.*, 2006; Kawa *et al.*, 2016). In agreement with the genotype-specific responses, the sensitivity of PR growth to low Pi seems to be highly species specific. For example, PRs of rice (*O. sativa*) show enhanced growth under Pi-deficient conditions (Shimizu *et al.*, 2004; Yi *et al.*, 2005), whereas in maize little to no enhancement was observed (Mollier and Pellerin, 1999; D. Wang *et al.*, 2017; Jia *et al.*, 2018).

Interestingly, the PR response to low Pi is linked to excessive iron accumulation that occurs in these conditions (Svistoonoff *et al.*, 2007). Under Pi starvation, roots secrete protons or organic acids that help to release anionic phosphate from cations in the soil, thereby also releasing and increasing the uptake of iron (Shahbaz *et al.*, 2006; Müller *et al.*, 2015; Balzergue *et al.*, 2017; Mora-Macías *et al.*, 2017). This disrupts the ratio of phosphate to iron in the root and causes apoplastic acidification, leading to peroxidase-dependent cell wall stiffening as well as accumulation of callose in the elongation zone (Balzergue *et al.*, 2017). This results in a reduced cell elongation rate followed by a progressive loss of meristematic cells (Ticconi *et al.*, 2004; Sánchez-Calderón *et al*., 2006; Lai *et al.*, 2007; Svistoonoff *et al.*, 2007). Other results suggest that accumulation of reactive oxygen species (ROS) under low Pi reduces PR length via cell death in the meristem (Pich *et al.*, 1994; Kampfenkel *et al.*, 1995; Caro and Puntarulo, 1996; Liszkay *et al.*, 2003; Chacón-López *et al.*, 2011). Furthermore, an intensive crosstalk between Pi and aluminium (Al) in plants can influence plant growth and metabolism (Clarkson, 1967). Accordingly, the levels of Pi can ameliorate the inhibitory effects of Al toxicity on root growth (Tan and Keltjens, 1990; Liao *et al.*, 2006; Sun *et al.*, 2008). Pi and Al sensing might use some common signalling mechanism to activate the response to both Pi deficiency and Al toxicity (Belal *et al.*, 2015; Dong *et al.*, 2017). Further studies are needed to better understand how the balance between Pi and metal levels in the meristem affects growth in PRs.

LRs play an important role in the acquisition of Pi in the soil by increasing the root system surface and supporting the solubilization of bound Pi (Lynch, 2007; Pérez-Torres *et al*., 2008; Kanno *et al.*, 2016). A common response to low Pi of plants is an increase in LR elongation rates (Drew, 1975; Bonser *et al.*, 1996; Williamson *et al.*, 2001; Linkohr *et al.*, 2002; Al-Ghazi *et al.*, 2003; Dai *et al.*, 2012). Under low Pi, dicots such as Arabidopsis and bean (*Phaseolus vulgaris*), as well as monocots such as barley, rice, and maize display increased branching to form tertiary and higher order LRs (Drew, 1975; Bonser *et al.*, 1996; Ticconi *et al.*, 2004; Negi *et al.*, 2016; Jia *et al.*, 2018). Interestingly, in Arabidopsis, the Pi starvation response is dependent on the age of LRs: in young LRs, the elongation is promoted under low Pi, whereas older LRs respond to Pi deficiency with reduced elongation (Nacry *et al.*, 2005; Sánchez-Calderón *et al*., 2006). It was suggested that different responses of young and old LRs to low Pi result from a redistribution of auxin in the root system (Nacry *et al.*, 2005); however, the exact mechanism remains elusive. The age-dependent response to Pi is somewhat reminiscent of the positional CK responses in LRs, but its potential interactions need to be assessed. Additionally, low Pi alters LR orientation in Arabidopsis towards a more vertical growth (Bai *et al.*, 2013). Altogether, the reduction of PR elongation and the change in elongation and growth direction of LRs result in a shallower RSA in response to phosphate starvation.

Double mutants of the PHOSPHATE TRANSPORTER 1;1 (PHT1;1) and PHT1;4 showed a faster LR elongation, but slower PR growth under low Pi (Shin *et al.*, 2004), suggesting a model in which the growth responses of LRs and PRs to phosphate deficiency are controlled by distinct pathways. This is supported by the observation that double mutants of the PHOSPHOLIPASE Dζ1 and 2 display enhanced elongation of the PRs and inhibition of the LRs under Pi starvation (Li, 2006). Further work is necessary to better understand how these transcription factors regulate RSA by transcriptional responses to Pi.

Finally, recent work in maize suggests that auxin and BR might play a role in Pi starvation. Altering the levels of the Arabidopsis homologue SERINE/THREONINE PROTEIN PHOSPHATASE 2A (ZmPP2AA1) 1 can change the response of PRs and LRs under phosphate starvation (J. Wang *et al.*, 2017). PP2A is known to control the transport of auxin in Arabidopsis (Rashotte *et al.*, 2001). Additionally, BR signalling has been shown to integrate low Pi and low iron signals to modulate PR growth responses (Singh *et al.*, 2018).These mechanisms should also be investigated in LRs to determine how the balance of Pi and iron regulates LR growth.

Overall, current data suggest that phosphate deficiency alters RSA by decreasing PR elongation and promoting LR elongation through transcriptional, hormonal, and developmental responses. The details of these mechanisms and the interactions between them remain to be elucidated.

### Nutrient availability distinctly alters developmental programmes in PRs and LRs

In addition to N and phosphate, the availability of other nutrients influences RSA. In Arabidopsis, sulfur starvation reduces the PR length but leads to slightly longer LRs. However, under prolonged sulfur deficiency, LR growth is also reduced (Gruber *et al.*, 2013). Plants with calcium deficiency produce a very shallow and highly branched root system in which the length of the PR is decreased and the elongation rate of LRs is increased (Gruber *et al.*, 2013). Potassium, manganese, and magnesium deficiency in the soil reduced the length of both PRs and LRs (Gruber *et al.*, 2013; Kellermeier *et al.*, 2014). Boron-deficient plants show reduced PR length, while LR growth is resistant to low boron in the soil (Gruber *et al.*, 2013). Conversely, excess boron leads to boron toxicity, resulting in short PRs and LRs. (Aquea *et al.*, 2012). Finally, mild iron deficiency elicits the opposite growth response, stimulating a slight increase in PR and LR length (Giehl *et al.*, 2012; Lešková *et al.*, 2017). On the other hand, a strong deficiency distinctly reduces the length of the LRs, but has no influence on the elongation of the PR (Lešková *et al.*, 2017). Overall, the accumulating evidence suggests that deficiencies in specific nutrients initiate distinct developmental programmes in PRs and LRs. Future work should address the interaction between these nutrient deficiencies and the transcriptional, hormonal, and cellular mechanisms through which they operate.

### The root system is altered in response to osmotic stress

Osmotic stress is here referred to as situations in which water availability for plants is limited, resulting in growth and developmental defects. Such conditions can arise from drought, excessive salt concentrations, chilling, and freezing. When water becomes limited, root systems explore the soil to reach deeper soil layers. A mild water stress can enhance PR growth in Populus, *Lespedeza davurica* (L.), several oak (*Quercus*) species, Arabidopsis, and soybean (*Glycine max*) (Van Der Weele *et al.*, 2000; Bogeat-Triboulot *et al.*, 2007; Yamaguchi *et al.*, 2010; Arend *et al.*, 2011; Kuster *et al.*, 2012; Xu *et al.*, 2012) and deep-rooted genotypes generally grew better under drought stress (Ho *et al.*, 2005). *Vicia faba* (bean) accessions from drier regions have a larger root system than those from well-irrigated, wet regions (Belachew *et al.*, 2018). Moreover, it has been shown that the PR tip can grow towards wetter soil and away from high osmolarity (Takahashi *et al.*, 2002), a phenomenon described as hydrotropism. Likewise, it has been shown in rice and Arabidopsis that the expression of the DEEPER ROOTING 1 (DRO1)/NEGATIVE GRAVITROPIC RESPONSE OF ROOTS 2 (NGR2)/LAZY 4 (LZY4) gene leads to plants with a steeper root system, thereby enhancing drought tolerance (Uga et al., 2013, 2015; Yoshihara and Spalding, 2017). The exact mechanism whereby roots sense drought and initiate the subsequent downstream signalling is not fully understood. However, it is known that ABA plays an important role in drought-responsive signalling; several studies demonstrated that drought conditions induce ABA production in roots. Interestingly, young PRs accumulate more ABA then older ones (Zhang and Davies, 1989; Zhang and Tardieu, 1996). ABA accumulation alters polar auxin transport rates in the root tip to stimulate cell elongation (Xu *et al.*, 2012; Rowe *et al.*, 2016; T. Wang *et al.*, 2017), suggesting a possible link between drought-induced ABA signalling and changes in auxin that are jointly integrated into RSA remodelling.

In Arabidopsis, elongation of LRs is significantly inhibited by drought (Xiong *et al.*, 2006), though the molecular mechanism of this response remains unclear. Genetic screens have identified the so-called *drought inhibition of lateral root growth* (*dig*) mutants with drought-resistant LR elongation (Xiong *et al.*, 2006), but the underlying gene remains to be revealed. Recent work suggests that the MYB96 transcription factor mediates an ABA–auxin crosstalk response specifically in LRs that are exposed to drought stress (Seo *et al.*, 2009).

Hyperosmotic conditions in the soil due to high concentrations of Na^+^ and other ions can also prevent plant absorption of water and nutrients (Ismail *et al.*, 2014). In Arabidopsis, PR and LR growth is reduced by high salinity, although the elongation of LRs shows stronger inhibition (Burssens *et al.*, 2000; Zolla *et al.*, 2010; Duan *et al.*, 2013; Kawa *et al.*, 2016). Similar to the response to drought stress, high salinity activates the ABA response pathway (Jia *et al.*, 2018), though during salt stress it may be restricted specifically to the endodermis of LRs (Duan *et al.*, 2013). In summary, osmotic stress operates through hormone signalling to modulate PR elongation and growth direction as well as LR growth. Future work should examine the role of osmotic stress in defining RSA via modulation of LR growth direction.

### Temperature responses in main and lateral roots are distinct

The growth, development, and productivity of plants are strongly influenced by the ambient temperature. Each plant species has a specific temperature range with optimal growth behaviour (Hatfield *et al.*, 2011; Hatfield and Prueger, 2015), and temperature alterations, particularly strong increases, can have dramatic consequences for plant survival. In most plant species, the rate of PR elongation increases linearly with rising temperature until a certain, species-specific threshold is reached, at which PR growth rate falls sharply as temperature is further increased (Al-ani and Hay, 1983; Sattelmacher *et al.*, 1990; Seiler, 1998; Nagel *et al.*, 2009). Several studies suggest that high temperature affects the PR growth in an auxin-dependent (Hanzawa *et al.*, 2013; Wang *et al.*, 2015; Fei *et al.*, 2017; Feraru *et al.*, 2019) and BR-dependent manner (Martins *et al.*, 2017; Sun *et al.*, 2019, Preprint). In contrast to PRs, LRs show a very wide range of different responses to heat across species. For example, LRs of rapeseed [*Brassica napus* (L.)] do not show any growth differences when grown under increased temperature (Nagel *et al.*, 2009). In sunflower (*Helianthus annuus*), temperatures below 20 °C and above 35 °C favour PR growth, while temperatures in the 20–35 °C range favour LR growth (Seiler, 1998). In potato (*Solanum tuberosum*), PR growth at high temperatures was considerably reduced compared with LR growth (Sattelmacher *et al.*, 1990). Unfortunately, mechanistic insights explaining the distinct responses in PRs and LRs are not yet available.

### Light modulates the root system architecture

Altering growth in response to light is key for plant survival. In addition to modulating shoot and flower development, light affects root morphology. Light inhibits PR growth in several mono- and dicotyledon plant species (Burström, 1960; Ohno, 1967; Wain *et al.*, 1968; Wilkins and Wain, 1974; Pilet and Ney, 1978) by reducing cell division activity and inhibiting cellular elongation (Ohno, 1967). This effect is dependent on the intensity and wavelength of the light (Ohno, 1967; Shen-miller, 1978). In Arabidopsis, the transcription factor LONG HYPOCOTYL5 (HY5) promotes the expression of negative regulators of auxin signalling, thereby linking hormone and light signalling pathways (Cluis *et al.*, 2004).

Comparably less is known about the influence of light on the growth of LRs. In Arabidopsis, the LRs of light-grown seedlings are longer when compared with dark-grown seedlings (Moni *et al.*, 2015). This effect seems to be dependent on the blue light receptor PHOTOTROPIN-1 (PHOT1) because LRs of *phot1* mutants displayed no difference under these conditions (Moni *et al.*, 2015). The authors suggest that PHOT1 plays a role in the elongation of LRs through the control of an auxin-related signalling pathway (Moni *et al.*, 2015). Future work should address how hormone signalling pathways alter developmental programmes of PRs and LRs in response to light.

### Main and lateral roots display distinct gravitropic set point angles

After germination, shoots must orient upwards to access light, while roots grow downward into the soil to access water and nutrients. PRs have an adaptive mechanism to resume downward growth should their orientation relative to the gravity vector be disturbed. After reorientation, starch-filled statoliths sediment to the lower side of columella cells and initiate a signal transduction pathway which harnesses second messengers such as inositol 1,4,5-triphosphate (IP3), Ca^2+^, and H^+^ (Su *et al.*, 2017). Through an as yet uncharacterized mechanism, statolith sedimentation and subsequent signalling result in the polarization of PIN-dependent auxin transport to the lower side of the root (Leitz *et al.*, 2009; Baldwin *et al.*, 2013). This process leads to higher auxin accumulation on the lower root flank, resulting in reduced growth on this side and bending towards gravity (Friml *et al.*, 2002; Kleine-Vehn *et al.*, 2010; Ogura *et al.*, 2019).

In contrast to PRs, LRs partially suppress positive gravitropic growth and maintain a gravitropic set point angle (GSA) that allows radial expansion of the root system (plagiotropism) (Digby and Firn, 1995). The hormonal signals modulating asymmetric growth in LRs have been recently elucidated. TIR1/AFB-Aux/IAA-ARF-dependent auxin signalling within the gravity-sensing cells is necessary to establish a GSA in the LRs (Roychoudhry *et al.*, 2013). Like PRs, LRs develop columella cells and amyloplasts in the root tips (Kiss *et al.*, 2002). In recently emerged (so-called stage I) LRs, PIN4 and PIN7 are strongly repressed when compared with the PR, and only PIN3 is transiently expressed in columella cells, presumably limiting the strength of auxin redistribution in (stage II) LRs that are establishing their GSA (Rosquete *et al.*, 2013; Ruiz Rosquete *et al.*, 2018). The subsequent repression of PIN3 after GSA establishment coincides with symmetric auxin signalling at the LR organ flank and symmetric elongation, maintaining the primary GSA (Rosquete *et al.*, 2013). While auxin promotes bending towards gravity during GSA establishment, CK functions as an LR-specific anti-gravitropic component by reducing cellular elongation and proliferation in the upper organ flank of emerged stage II LRs. In this way, CK attenuates LR bending towards gravity and promotes the radial expansion of the root system (Waidmann *et al.*, 2019).

Though hormonal mechanisms tuning angular LR growth have been recently explored, many open questions remain about how developmental and environmental signals modulate hormone signals and transcriptional cascades to modulate angular LR growth. In particular, it will be crucial to uncover how these mechanisms interact to produce distinct directional growth responses in PRs and LRs.

## Conclusion

**Fig. 2.**
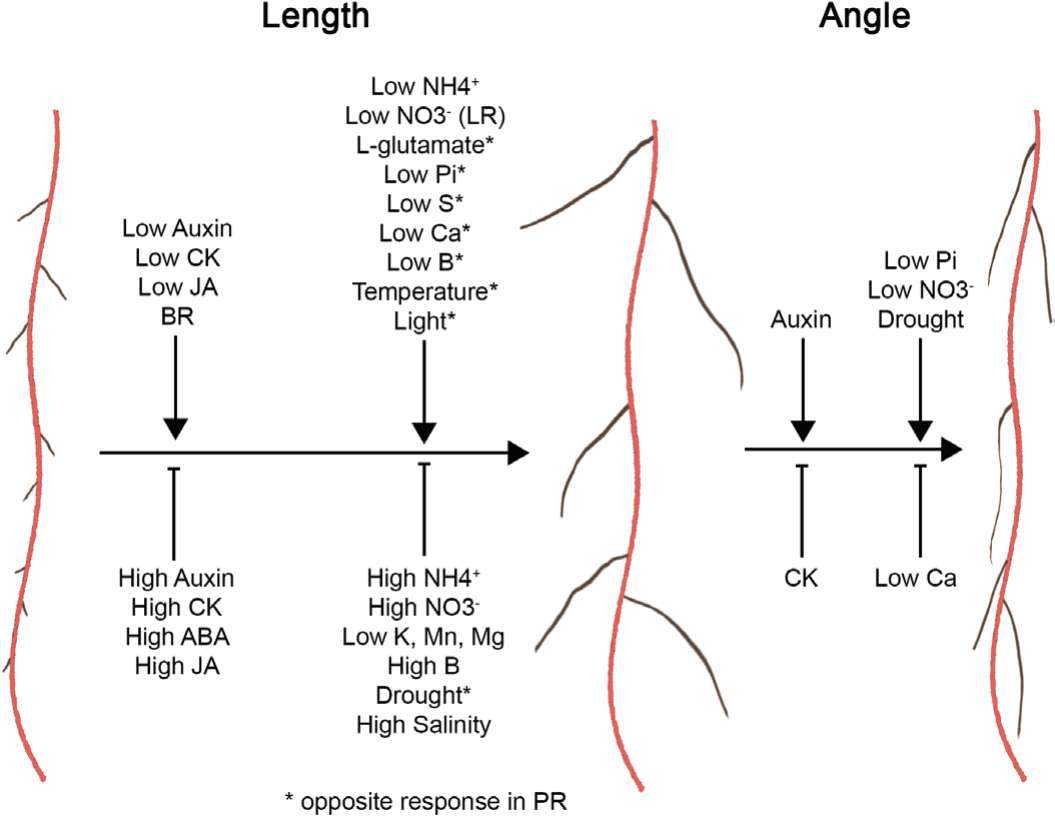
Many endogenous and exogenous signals alter lateral root (LR) development in Arabidopsis. Various external and internal stimuli can influence the length and angle of LRs. The asterisks indicate an opposite response in PRs.

RSA is highly dynamic, responding to external and endogenous cues to adapt to constantly fluctuating conditions (Fig. 2). The mechanisms that control PR and LR growth have been mainly studied separately, reinforcing the incorrect assumption that the mechanisms regulating PR and LR growth are nearly identical. Thanks to the effort of a rapidly growing root community, it becomes increasingly clear that PRs and LRs share common regulatory mechanisms, which are distinctly and combinatorially interpreted to enable distinct growth behaviours. In particular, environmental cues initiate unique growth outcomes in LRs and PRs. This enables plants to dynamically modify RSA to explore the soil and find nutrient-rich patches or reach deeper, water-bearing soil formations.

Most of the here described responses are often dependent on the developmental context of the root organ. While this may be more apparent when studying LRs, most studies on PRs concentrate on a single time point. Hence, a better understanding of age-dependent responses in primary and lateral roots is needed to substantiate which of the responses of PRs and LRs underlie truly distinct developmental programmes. A challenging step will be to understand the transcriptional and post-translational mechanisms that define the distinct nature of PRs and LRs because they are likely to be key to understanding the distinct hormonal, physiological, and developmental outputs.
